# 1,3-Bis{(*E*)-[4-(di­methyl­amino)­benzyl­idene]amino}­propan-2-ol: chain structure formation *via* an O—H⋯N hydrogen bond

**DOI:** 10.1107/S2056989017006429

**Published:** 2017-05-05

**Authors:** Augusto Rivera, Ingrid Miranda-Carvajal, Jaime Ríos-Motta, Michael Bolte

**Affiliations:** a Universidad Nacional de Colombia, Sede Bogotá, Facultad de Ciencias, Departamento de Química, Cra 30 No. 45-03, Bogotá, Código Postal 111321, Colombia; b Institut für Anorganische Chemie, J. W. Goethe-Universität Frankfurt, Max-von-Laue-Str. 7, 60438 Frankfurt/Main, Germany

**Keywords:** crystal structure, Schiff bases, supra­molecular chain, super-structure, pseudo-symmetry

## Abstract

The asymmetric unit of the title compound, C_21_H_28_N_4_O, consists of two unique mol­ecules linked by an O—H⋯N hydrogen bond. The conformation of both C=N bonds is *E* and the azomethine functional groups lie close to the plane of their associated benzene rings in each of the independent mol­ecules. The dihedral angles between the two benzene rings are 83.14 (4) and 75.45 (4)°. The plane of the one of the N(CH_3_)_2_ units is twisted away from the benzene ring by 18.8 (2)°, indicating loss of conjugation between the lone electron pair and the benzene ring. In the crystal structure, O—H⋯N hydrogen bonds together with C—H⋯O hydrogen bonds link neighbouring supra­molecular dimers into a three-dimensional network.

## Abstract 2

The mol­ecular and crystal structure of the title Schiff base derivative is reported. O—H⋯N hydrogen bonds link mol­ecules into a supra­molecular chain along *a*.

## Chemical context   

Schiff bases play important roles in the development of coordination chemistry related to catalysis, enzymatic reactions, and supra­molecular architectures. Crystal structures of Schiff bases derived from substituted benzaldehydes and 1,3-di­amino­propan-2-ol have been reported earlier (Azam, Warad, Al-Resayes *et al.*, 2012 ▸; Azam, Hussain *et al.*, 2012 ▸; Rivera *et al.*, 2016*b* ▸, 2017 ▸; Elmali, 2000 ▸). The title compound, (I), acts as an important raw material for the synthesis of Schiff base complexes. As an extension of our work on the synthesis and structural characterization of such Schiff base compounds, the crystal structure of the title compound is reported here.
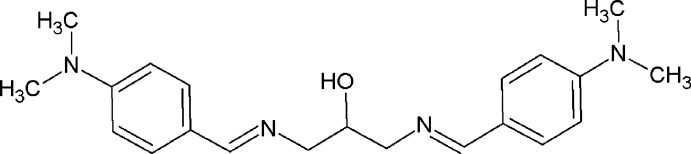


## Structural commentary   

**Figure 1:**
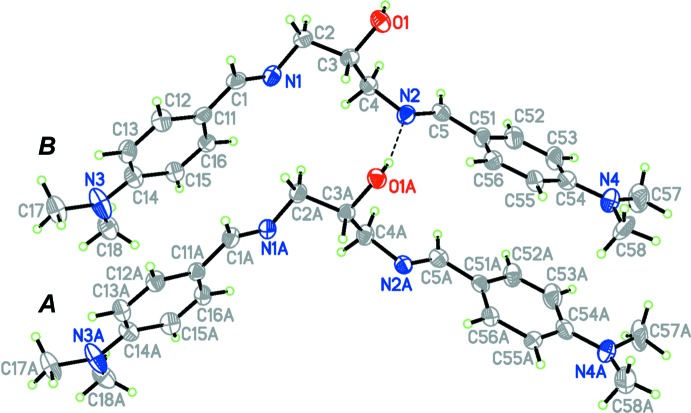
The structure of the independent mol­ecules *A* and *B*, showing the atom-labelling scheme. Displacement ellipsoids are drawn at the 50% probability level for non-H atoms.

**Figure 2:**
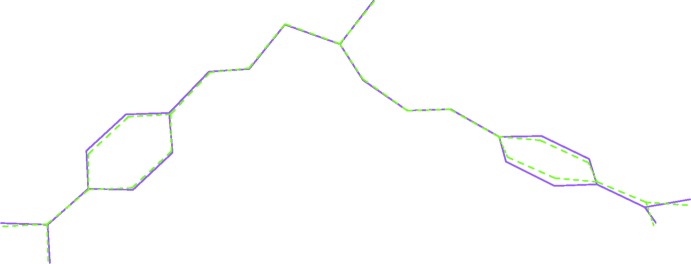
The structural overlay of the independent mol­ecules *A* (green dashed) and *B* (purple) of the title compound.

The title compound crystallizes with two unique mol­ecules in the asymmetric unit. The conformers, labeled *A* and *B*, are shown in Fig. 1 ▸. Each mol­ecule comprises a 1,3-di­amino-2-hy­droxy­propane bridge symmetrically substituted at the 1 and 3 positions by 4-(di­methyl­amino)­phen­yl]methyl­idene units. The conformational differences between the two mol­ecules are extremely small, resulting in a superstructural motif. The two mol­ecules are related by translation along the *a*-axis direction. A structural overlay of the two independent mol­ecules (r.m.s. deviation for fitting all non-H atoms = 0.097 Å) is shown in Fig. 2 ▸. The disposition of the residues attached to the N2*A* and N2 positions can be described by the torsion angles N2*A*—C5*A*—C51*A*—C56*A* [−9.9 (11) in mol­ecule *A*] and N2—C5—C51—C56 [−14.9 (11)° in mol­ecule *B*]. The two outer aromatic rings (C11–C16 and C51–C56) are inclined to one another by 83.14 (4)° in mol­ecule *A* and 75.45 (4)° in mol­ecule *B*.

Bond distances and angles in the benzene rings are not unusual and compare well, both between the two independent mol­ecules and with those observed in related systems (see for example: Rivera *et al.*, 2016*b* ▸). The values for the azomethine C=N bond distances in the two mol­ecules [1.275 (8) and 1.272 (8) in mol­ecule *A* and 1.271 (8) and 1.269 (8) Å in mol­ecule *B*] and the corresponding inter­nal angles at the nitro­gen atom [C1*A*—N1*A*—C2*A* = 117.7 (6) and C5*A*—N2*A*—C4*A* = 117.7 (6) in mol­ecule *A* and C1—N1—C2 = 117.5 (6) and C5—N2—C4 = 117.6 (6) in mol­ecule *B*] also agree with those reported in the literature for similar compounds (Rivera *et al.*, 2016*b* ▸) and are consistent with C=N double bonding. In both mol­ecules, the azomethine groups adopt an *E*,*E* conformation, as can be seen from the torsion angles C2*A*—N1*A*—C1*A*—C11*A* = 177.8 (6)° and C4*A*—N2*A*—C5*A*—C51*A* = 179.9 (6)° in mol­ecule *A* and C2—N1—C1—C11 = 178.6 (6)° and C4—N2—C5—C51 = 177.0 (6) in mol­ecule *B.*

The two di­methyl­amino substituents in mol­ecule *B* are essentially coplanar with the benzene rings to which they are bound with torsion angles C17—N3—C14—C13 = −3.1 (11)° and C57—N4—C54—C53 = −2.9 (11)° and with dihedral angles between the NMe_2_ plane and the benzene ring of 0.57 (2) and 4.60 (2)°, respectively, whilst in mol­ecule *A* the corresponding torsional angles C17*A*—N3*A*—C14*A*—C13*A* and C57*A*—N4*A*—C54*A*—C53*A* are 2.2 (11) and 8.3 (10)°, respectively. The dihedral angles between the two di­methyl­amino groups (N3*A* and N4*A*) and the benzene rings are 5.09 (22) and 18.8 (2)° respectively, indicating that the lone electron pair of the N4*A* atom may not be completely conjugated with the benzene ring (C51*A*–C56*A*).

## Supra­molecular features   

**Figure 3:**
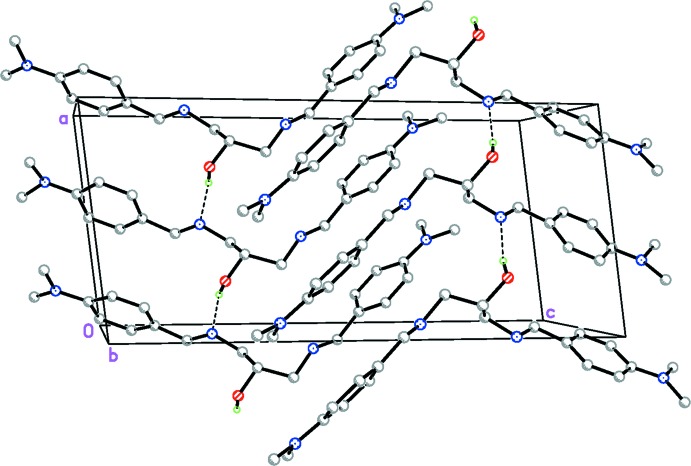
Crystal packing of the title compound, indicating the O—H⋯N hydrogen bonds (dashed lines), which result in chains along the *a*-axis direction.

Through O—H⋯N hydrogen-bonding inter­actions [2.863 (7) Å] between O1*A*—H1*A* and the nitro­gen N2 (Table 1 ▸), the two independent mol­ecules inter­act to form *C*(5) chains running along the *a* axis (Fig. 3 ▸). The chains are linked into a three-dimensional framework by a pair of weaker inter­molecular C57—H57*B*⋯O1^ii^ and C57*A*—H57*E*⋯O1*A* ^iii^ hydrogen bonds (Table 1 ▸).

## Database survey   

A search in the Cambridge Crystallographic Database (Groom *et al.*, 2016 ▸) for the fragment 1,3-bis­[(benzyl­idene)amino]­propan-2-ol yielded the following structures: *N*,*N*′-[(2-hy­droxy-1,3-propanedi­yl)bis­(nitrilo­methylyl­idene-2,1-phenyl­ene)]bis­(4-methyl­benzene­sulfonamide) (Popov *et al.*, 2009 ▸), 2,2′-[(2-hy­droxy­propane-1,3-di­yl)bis­(nitrilo­meth­yl­yl­idene)]diphenol (Azam, Hussain *et al.*, 2012 ▸), 1,3-bis­(2-hy­droxy-5-bromo­salicyl­idene­amine)­propan-2-ol (Elmali, 2000 ▸), 1,3-bis­[(*E*)-(2-chloro­benzyl­idene)amino]­propan-2-ol (Azam, Warad, Al-Resayes *et al.*, 2012 ▸) and 1,3-bis­[(4-meth­oxy­benzyl­idene)amino]­propan-2-ol (Rivera *et al.* 2016*b* ▸). In each of these structures, the N=C double bonds adopt *E* conformations.

## Synthesis and crystallization   

The title compound was prepared as described by (Rivera *et al.* 2016*a* ▸). The crude product was recrystallized from diethyl ether solution by slow evaporation of the solvent, giving colorless crystals suitable for X–ray diffraction (m.p. 396.8–398 K; yield 40%).

## Refinement   

**Figure 4:**
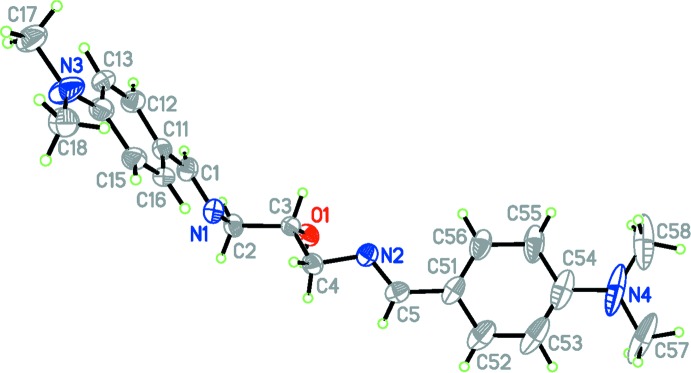
Perspective view of the mol­ecule if the structure is refined in a cell with the *a* axis halved.

**Figure 5:**
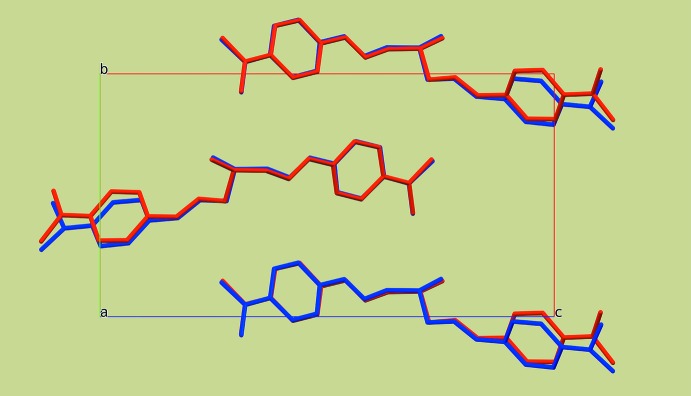
Partial packing diagram of the title compound with one mol­ecule shifted by *x* = ½, *y* = 0, *z* = 0, showing similarities and differences between the two mol­ecules.

Crystal data, data collection and structure refinement details are summarized in Table 2 ▸. The coordinates of the hydroxyl H atom were refined with *U*_iso_(H) = 1.5*U*_eq_(O). The remaining H atoms were positioned geometrically and allowed to ride on their parent atoms, with *d*(C—H) = 0.95 Å for aromatic and azomethine atoms, *d*(C—H) = 0.98 Å for methyl, *d*(C—H) = 0.99 Å for methyl­ene, *d*(C—H) = 1.00 Å for tertiary CH. The *U*_iso_(H) values were constrained to 1.5*U*_eq_(C_meth­yl_) or 1.2*U*_eq_(C) for the remaining H atoms. The structure shows signs of a superstructure. The two mol­ecules are related by a translation of 1/2 along the *a* axis. However, if the structure is refined in a cell with the *a* axis halved, the displacement parameters of one NMe_2_ group and some of the C atoms of the phenyl ring to which this group is attached are significantly enlarged (Fig. 4 ▸). Shifting one mol­ecule by ½ in the *a*-axis direction, it becomes obvious how similar the two mol­ecules are. Nevertheless, there are small differences in their overall conformation (Fig. 5 ▸). As a result of that, we opted to refine the structure using the larger unit cell with two mol­ecules in the asymmetric unit.

## Supplementary Material

Crystal structure: contains datablock(s) I. DOI: 10.1107/S2056989017006429/sj5528sup1.cif

Structure factors: contains datablock(s) I. DOI: 10.1107/S2056989017006429/sj5528Isup2.hkl

CCDC reference: 1547455

Additional supporting information:  crystallographic information; 3D view; checkCIF report

## Figures and Tables

**Table 1:** Hydrogen-bond geometry (Å, °)

*D*—H⋯*A*	*D*—H	H⋯*A*	*D*⋯*A*	*D*—H⋯*A*
O1—H1⋯N2*A* ^i^	0.84 (8)	2.06 (8)	2.889 (7)	170 (7)
O1*A*—H1*A*⋯N2	0.84 (8)	2.04 (8)	2.863 (7)	166 (7)
C57—H57*B*⋯O1^ii^	0.98	2.53	3.171 (9)	123
C57*A*—H57*E*⋯O1*A* ^iii^	0.98	2.36	3.211 (8)	145

Symmetry codes: (i) 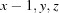; (ii) 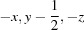; (iii) 
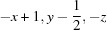
.

**Table 2:** Experimental details

Crystal data	
Chemical formula	C_21_H_28_N_4_O
*M*_r_	352.47
Crystal system, space group	Monoclinic, *P*2_1_
Temperature (K)	173
*a*, *b*, *c* (Å)	9.1456 (10), 10.5860 (8), 19.974 (2)
β (°)	97.110 (9)
*V* (Å^3^)	1918.9 (3)
*Z*	4
Radiation type	Mo *K*α
μ (mm^−1^)	0.08
Crystal size (mm)	0.22 × 0.03 × 0.03

Data collection	
Diffractometer	STOE IPDS II two-circle
Absorption correction	Multi-scan (*X-AREA*; Stoe & Cie, 2001 ▸)
*T*_min_, *T*_max_	0.426, 1.000
No. of measured, independent and observed [*I* > 2σ(*I*)] reflections	16633, 6852, 3688
*R*_int_	0.069
(sin θ/λ)_max_ (Å^−1^)	0.609

Refinement	
*R*[*F* ^2^ > 2σ(*F* ^2^)], *wR*(*F* ^2^), *S*	0.063, 0.156, 0.93
No. of reflections	6852
No. of parameters	483
No. of restraints	1
H-atom treatment	H atoms treated by a mixture of independent and constrained refinement
Δρ_max_, Δρ_min_ (e Å^−3^)	0.17, −0.22

Computer programs: *X-AREA* (Stoe & Cie, 2001 ▸), *SHELXS* (Sheldrick, 2008 ▸), *SHELXL2014/7* (Sheldrick, 2015 ▸) and *XP* in *SHELXTL-Plus* (Sheldrick, 2008 ▸).

## supplementary crystallographic information

### Crystal data

**Table:**

C_21_H_28_N_4_O	*F*(000) = 760
*M**_r_* = 352.47	*D*_x_ = 1.220 Mg m^−^^3^
Monoclinic, *P*2_1_	Mo *K*α radiation, λ = 0.71073 Å
*a* = 9.1456 (10) Å	Cell parameters from 8852 reflections
*b* = 10.5860 (8) Å	θ = 3.2–25.9°
*c* = 19.974 (2) Å	µ = 0.08 mm^−^^1^
β = 97.110 (9)°	*T* = 173 K
*V* = 1918.9 (3) Å^3^	Needle, colourless
*Z* = 4	0.22 × 0.03 × 0.03 mm

### Data collection

**Table:**

STOE IPDS II two-circle-diffractometer	3688 reflections with *I* > 2σ(*I*)
Radiation source: Genix 3D IµS microfocus X-ray source	*R*_int_ = 0.069
ω scans	θ_max_ = 25.7°, θ_min_ = 3.2°
Absorption correction: multi-scan (X-Area; Stoe & Cie, 2001)	*h* = −11→11
*T*_min_ = 0.426, *T*_max_ = 1.000	*k* = −11→12
16633 measured reflections	*l* = −24→24
6852 independent reflections	

### Refinement

**Table:**

Refinement on *F*^2^	1 restraint
Least-squares matrix: full	Hydrogen site location: mixed
*R*[*F*^2^ > 2σ(*F*^2^)] = 0.063	H atoms treated by a mixture of independent and constrained refinement
*wR*(*F*^2^) = 0.156	*w* = 1/[σ^2^(*F*_o_^2^) + (0.0713*P*)^2^] where *P* = (*F*_o_^2^ + 2*F*_c_^2^)/3
*S* = 0.93	(Δ/σ)_max_ < 0.001
6852 reflections	Δρ_max_ = 0.17 e Å^−^^3^
483 parameters	Δρ_min_ = −0.22 e Å^−^^3^

### Special details

**Table:**

Geometry. All esds (except the esd in the dihedral angle between two l.s. planes) are estimated using the full covariance matrix. The cell esds are taken into account individually in the estimation of esds in distances, angles and torsion angles; correlations between esds in cell parameters are only used when they are defined by crystal symmetry. An approximate (isotropic) treatment of cell esds is used for estimating esds involving l.s. planes.

### Fractional atomic coordinates and isotropic or equivalent isotropic displacement parameters (Å^2^)

**Table:**

	*x*	*y*	*z*	*U*_iso_*/*U*_eq_	
O1	−0.2650 (5)	0.6469 (4)	0.2463 (2)	0.0338 (11)	
H1	−0.333 (9)	0.594 (7)	0.243 (4)	0.051*	
N1	−0.0704 (6)	0.5677 (5)	0.4156 (3)	0.0337 (13)	
N2	−0.0059 (6)	0.4851 (5)	0.2177 (3)	0.0325 (13)	
N3	0.4143 (8)	0.5502 (6)	0.6804 (3)	0.056 (2)	
N4	0.1885 (8)	0.4185 (7)	−0.0846 (3)	0.0525 (18)	
C1	−0.0252 (7)	0.6501 (6)	0.4596 (3)	0.0318 (15)	
H1B	−0.0717	0.7305	0.4578	0.038*	
C2	−0.1945 (7)	0.6018 (7)	0.3656 (3)	0.0329 (15)	
H2A	−0.2318	0.6862	0.3764	0.040*	
H2B	−0.2752	0.5401	0.3672	0.040*	
C3	−0.1482 (7)	0.6038 (6)	0.2949 (3)	0.0280 (14)	
H3	−0.0654	0.6657	0.2953	0.034*	
C4	−0.0905 (7)	0.4759 (6)	0.2754 (3)	0.0295 (14)	
H4A	−0.0268	0.4401	0.3145	0.035*	
H4B	−0.1745	0.4177	0.2640	0.035*	
C5	−0.0408 (7)	0.4112 (6)	0.1683 (3)	0.0291 (14)	
H5	−0.1163	0.3509	0.1720	0.035*	
C11	0.0959 (7)	0.6257 (6)	0.5131 (3)	0.0286 (14)	
C12	0.1407 (8)	0.7210 (6)	0.5603 (3)	0.0349 (16)	
H12	0.0966	0.8023	0.5552	0.042*	
C13	0.2491 (8)	0.6979 (6)	0.6143 (3)	0.0346 (16)	
H13	0.2791	0.7642	0.6451	0.041*	
C14	0.3151 (8)	0.5775 (6)	0.6241 (3)	0.0340 (16)	
C15	0.2724 (7)	0.4838 (6)	0.5751 (3)	0.0313 (15)	
H15	0.3170	0.4027	0.5792	0.038*	
C16	0.1666 (7)	0.5092 (6)	0.5215 (3)	0.0282 (14)	
H16	0.1408	0.4448	0.4891	0.034*	
C17	0.4596 (10)	0.6471 (7)	0.7298 (4)	0.050 (2)	
H17A	0.5096	0.7152	0.7084	0.075*	
H17B	0.5272	0.6106	0.7667	0.075*	
H17C	0.3728	0.6810	0.7478	0.075*	
C18	0.4822 (8)	0.4275 (7)	0.6890 (4)	0.0458 (18)	
H18A	0.4071	0.3642	0.6956	0.069*	
H18B	0.5574	0.4287	0.7285	0.069*	
H18C	0.5283	0.4061	0.6487	0.069*	
C51	0.0284 (8)	0.4128 (7)	0.1056 (3)	0.0330 (16)	
C52	0.0002 (8)	0.3146 (7)	0.0598 (3)	0.0431 (17)	
H52	−0.0583	0.2455	0.0713	0.052*	
C53	0.0546 (9)	0.3135 (8)	−0.0025 (3)	0.0458 (19)	
H53	0.0343	0.2439	−0.0322	0.055*	
C54	0.1384 (8)	0.4140 (8)	−0.0211 (4)	0.0390 (17)	
C55	0.1671 (8)	0.5154 (7)	0.0254 (3)	0.0376 (17)	
H55	0.2227	0.5861	0.0138	0.045*	
C56	0.1155 (8)	0.5119 (7)	0.0867 (3)	0.0351 (16)	
H56	0.1396	0.5792	0.1175	0.042*	
C57	0.1555 (11)	0.3106 (9)	−0.1292 (4)	0.066 (3)	
H57A	0.1979	0.2340	−0.1072	0.099*	
H57B	0.1980	0.3246	−0.1713	0.099*	
H57C	0.0485	0.3008	−0.1392	0.099*	
C58	0.2838 (10)	0.5179 (9)	−0.1020 (4)	0.059 (2)	
H58A	0.2329	0.5992	−0.1009	0.089*	
H58B	0.3103	0.5030	−0.1474	0.089*	
H58C	0.3734	0.5193	−0.0695	0.089*	
O1A	0.2364 (5)	0.6551 (4)	0.2491 (2)	0.0307 (10)	
H1A	0.175 (9)	0.598 (7)	0.236 (4)	0.046*	
N1A	0.4307 (6)	0.5729 (5)	0.4178 (2)	0.0321 (13)	
N2A	0.4841 (6)	0.4805 (5)	0.2205 (3)	0.0272 (12)	
N3A	0.9201 (8)	0.5491 (6)	0.6802 (3)	0.0528 (18)	
N4A	0.6733 (7)	0.3641 (7)	−0.0786 (3)	0.0493 (16)	
C1A	0.4784 (8)	0.6543 (6)	0.4623 (3)	0.0320 (15)	
H1A1	0.4335	0.7353	0.4603	0.038*	
C2A	0.3069 (7)	0.6088 (6)	0.3686 (3)	0.0301 (14)	
H2A1	0.2727	0.6943	0.3792	0.036*	
H2A2	0.2244	0.5490	0.3710	0.036*	
C3A	0.3525 (7)	0.6076 (6)	0.2969 (3)	0.0267 (14)	
H3A	0.4386	0.6658	0.2967	0.032*	
C4A	0.4027 (8)	0.4766 (6)	0.2788 (3)	0.0314 (15)	
H4A1	0.4663	0.4404	0.3178	0.038*	
H4A2	0.3156	0.4210	0.2688	0.038*	
C5A	0.4427 (8)	0.4067 (6)	0.1714 (3)	0.0295 (15)	
H5A	0.3609	0.3534	0.1759	0.035*	
C11A	0.5983 (7)	0.6298 (6)	0.5160 (3)	0.0261 (14)	
C12A	0.6474 (7)	0.7222 (6)	0.5632 (3)	0.0317 (15)	
H12A	0.6067	0.8047	0.5582	0.038*	
C13A	0.7528 (8)	0.6973 (6)	0.6165 (3)	0.0345 (16)	
H13A	0.7825	0.7622	0.6481	0.041*	
C14A	0.8175 (8)	0.5775 (6)	0.6252 (3)	0.0336 (15)	
C15A	0.7757 (8)	0.4861 (6)	0.5758 (3)	0.0355 (16)	
H15A	0.8212	0.4053	0.5791	0.043*	
C16A	0.6686 (7)	0.5124 (6)	0.5223 (3)	0.0295 (14)	
H16A	0.6423	0.4492	0.4892	0.035*	
C17A	0.9607 (10)	0.6408 (7)	0.7321 (4)	0.052 (2)	
H17D	1.0069	0.7135	0.7128	0.077*	
H17E	1.0305	0.6029	0.7676	0.077*	
H17F	0.8725	0.6686	0.7512	0.077*	
C18A	0.9840 (8)	0.4245 (7)	0.6888 (4)	0.0457 (18)	
H18D	0.9081	0.3642	0.6985	0.069*	
H18E	1.0637	0.4255	0.7264	0.069*	
H18F	1.0235	0.3994	0.6474	0.069*	
C51A	0.5096 (8)	0.3966 (6)	0.1087 (3)	0.0304 (16)	
C52A	0.4621 (8)	0.3029 (6)	0.0628 (3)	0.0386 (16)	
H52A	0.3901	0.2446	0.0740	0.046*	
C53A	0.5148 (9)	0.2908 (7)	0.0017 (4)	0.0457 (18)	
H53A	0.4794	0.2247	−0.0282	0.055*	
C54A	0.6205 (9)	0.3753 (8)	−0.0169 (3)	0.0409 (19)	
C55A	0.6708 (8)	0.4707 (8)	0.0289 (3)	0.0403 (17)	
H55A	0.7434	0.5288	0.0180	0.048*	
C56A	0.6136 (8)	0.4806 (7)	0.0914 (3)	0.0371 (17)	
H56A	0.6475	0.5462	0.1219	0.045*	
C57A	0.6067 (10)	0.2760 (8)	−0.1289 (3)	0.063 (3)	
H57D	0.6115	0.1904	−0.1100	0.095*	
H57E	0.6600	0.2786	−0.1685	0.095*	
H57F	0.5034	0.2993	−0.1421	0.095*	
C58A	0.7565 (10)	0.4673 (10)	−0.1032 (4)	0.065 (3)	
H58D	0.6943	0.5429	−0.1090	0.097*	
H58E	0.7881	0.4437	−0.1466	0.097*	
H58F	0.8433	0.4850	−0.0706	0.097*	

### Atomic displacement parameters (Å^2^)

**Table:**

	*U*^11^	*U*^22^	*U*^33^	*U*^12^	*U*^13^	*U*^23^
O1	0.038 (3)	0.032 (3)	0.031 (2)	−0.002 (2)	−0.002 (2)	0.0081 (19)
N1	0.038 (3)	0.036 (3)	0.028 (3)	0.002 (2)	0.009 (3)	−0.001 (2)
N2	0.034 (3)	0.034 (3)	0.030 (3)	0.006 (2)	0.006 (2)	0.001 (2)
N3	0.081 (5)	0.032 (4)	0.047 (4)	−0.005 (3)	−0.027 (4)	0.001 (3)
N4	0.051 (4)	0.078 (5)	0.030 (3)	0.005 (4)	0.009 (3)	−0.013 (3)
C1	0.029 (4)	0.032 (4)	0.035 (4)	0.009 (3)	0.007 (3)	0.000 (3)
C2	0.028 (4)	0.041 (4)	0.031 (3)	0.003 (3)	0.007 (3)	0.001 (3)
C3	0.028 (3)	0.026 (3)	0.030 (3)	0.001 (3)	0.005 (3)	0.000 (2)
C4	0.030 (3)	0.032 (3)	0.027 (3)	−0.005 (3)	0.004 (3)	0.006 (3)
C5	0.027 (3)	0.028 (3)	0.031 (3)	−0.002 (3)	−0.002 (3)	−0.002 (3)
C11	0.026 (3)	0.032 (3)	0.030 (3)	−0.002 (3)	0.012 (3)	−0.001 (3)
C12	0.043 (4)	0.025 (3)	0.036 (4)	0.001 (3)	0.002 (3)	0.002 (3)
C13	0.044 (4)	0.026 (3)	0.034 (4)	−0.008 (3)	0.006 (3)	−0.008 (3)
C14	0.040 (4)	0.033 (4)	0.028 (3)	−0.009 (3)	0.001 (3)	0.008 (3)
C15	0.036 (4)	0.024 (3)	0.033 (3)	−0.003 (3)	0.003 (3)	0.001 (2)
C16	0.027 (3)	0.027 (3)	0.031 (3)	−0.005 (3)	0.006 (3)	−0.004 (2)
C17	0.064 (5)	0.046 (4)	0.037 (4)	−0.012 (4)	−0.010 (4)	−0.002 (3)
C18	0.054 (5)	0.043 (4)	0.037 (4)	−0.004 (4)	−0.005 (3)	0.009 (3)
C51	0.038 (4)	0.035 (4)	0.025 (3)	0.013 (3)	0.002 (3)	−0.005 (3)
C52	0.050 (4)	0.044 (4)	0.035 (4)	0.004 (3)	0.003 (3)	0.001 (3)
C53	0.054 (4)	0.052 (4)	0.030 (3)	0.009 (4)	0.000 (3)	−0.018 (3)
C54	0.033 (4)	0.052 (4)	0.032 (3)	0.007 (3)	0.001 (3)	−0.004 (3)
C55	0.038 (4)	0.047 (4)	0.027 (3)	0.001 (3)	−0.001 (3)	−0.004 (3)
C56	0.031 (4)	0.044 (4)	0.031 (3)	−0.004 (3)	0.003 (3)	−0.008 (3)
C57	0.086 (6)	0.073 (6)	0.038 (4)	0.029 (5)	0.008 (4)	−0.017 (4)
C58	0.054 (5)	0.088 (6)	0.041 (4)	0.015 (4)	0.030 (4)	0.012 (4)
O1A	0.031 (3)	0.035 (3)	0.026 (2)	0.000 (2)	0.0010 (19)	0.0069 (18)
N1A	0.032 (3)	0.038 (3)	0.026 (3)	0.002 (2)	0.003 (2)	−0.003 (2)
N2A	0.028 (3)	0.025 (3)	0.027 (3)	0.003 (2)	0.001 (2)	−0.002 (2)
N3A	0.068 (5)	0.037 (4)	0.044 (3)	−0.004 (3)	−0.030 (3)	0.003 (3)
N4A	0.046 (4)	0.074 (4)	0.029 (3)	0.004 (3)	0.011 (3)	−0.012 (3)
C1A	0.037 (4)	0.033 (4)	0.027 (4)	0.002 (3)	0.007 (3)	−0.005 (3)
C2A	0.032 (4)	0.033 (4)	0.025 (3)	−0.002 (3)	0.007 (3)	−0.001 (3)
C3A	0.029 (3)	0.027 (3)	0.025 (3)	−0.003 (3)	0.005 (3)	0.000 (2)
C4A	0.039 (4)	0.029 (3)	0.026 (3)	0.005 (3)	0.005 (3)	0.003 (3)
C5A	0.032 (4)	0.023 (3)	0.032 (3)	0.006 (3)	0.001 (3)	0.006 (2)
C11A	0.028 (3)	0.027 (3)	0.023 (3)	−0.001 (3)	0.004 (3)	0.000 (2)
C12A	0.037 (4)	0.025 (3)	0.034 (3)	0.003 (3)	0.004 (3)	−0.005 (3)
C13A	0.040 (4)	0.035 (4)	0.027 (3)	−0.005 (3)	−0.002 (3)	−0.002 (3)
C14A	0.031 (4)	0.032 (4)	0.036 (4)	−0.002 (3)	−0.003 (3)	0.000 (3)
C15A	0.045 (4)	0.024 (3)	0.037 (4)	−0.004 (3)	0.004 (3)	0.003 (3)
C16A	0.035 (4)	0.027 (3)	0.026 (3)	−0.008 (3)	0.004 (3)	−0.001 (2)
C17A	0.064 (5)	0.044 (4)	0.040 (4)	−0.007 (4)	−0.021 (4)	−0.003 (3)
C18A	0.046 (4)	0.040 (4)	0.047 (4)	−0.002 (4)	−0.014 (3)	0.015 (3)
C51A	0.027 (3)	0.033 (4)	0.029 (3)	0.006 (3)	−0.004 (3)	−0.003 (2)
C52A	0.052 (4)	0.031 (3)	0.031 (3)	0.005 (3)	0.003 (3)	−0.006 (3)
C53A	0.060 (5)	0.042 (4)	0.034 (3)	0.008 (4)	0.001 (3)	−0.008 (3)
C54A	0.036 (4)	0.057 (5)	0.028 (3)	0.024 (4)	−0.005 (3)	−0.014 (3)
C55A	0.027 (3)	0.065 (5)	0.029 (3)	0.001 (3)	0.006 (3)	−0.007 (3)
C56A	0.032 (4)	0.053 (4)	0.026 (3)	0.002 (3)	0.001 (3)	−0.008 (3)
C57A	0.090 (6)	0.074 (6)	0.024 (3)	0.038 (5)	0.001 (4)	−0.017 (3)
C58A	0.059 (5)	0.097 (7)	0.037 (4)	0.021 (5)	0.005 (4)	0.001 (4)

### Geometric parameters (Å, º)

**Table:**

O1—C3	1.426 (7)	O1A—C3A	1.429 (7)
O1—H1	0.84 (8)	O1A—H1A	0.84 (8)
N1—C1	1.271 (8)	N1A—C1A	1.275 (8)
N1—C2	1.461 (8)	N1A—C2A	1.454 (8)
N2—C5	1.269 (8)	N2A—C5A	1.272 (8)
N2—C4	1.470 (8)	N2A—C4A	1.460 (8)
N3—C14	1.386 (9)	N3A—C14A	1.388 (8)
N3—C18	1.440 (10)	N3A—C17A	1.434 (9)
N3—C17	1.448 (9)	N3A—C18A	1.444 (10)
N4—C54	1.400 (9)	N4A—C54A	1.383 (9)
N4—C58	1.436 (11)	N4A—C57A	1.449 (10)
N4—C57	1.458 (11)	N4A—C58A	1.452 (12)
C1—C11	1.463 (9)	C1A—C11A	1.459 (8)
C1—H1B	0.9500	C1A—H1A1	0.9500
C2—C3	1.523 (9)	C2A—C3A	1.542 (8)
C2—H2A	0.9900	C2A—H2A1	0.9900
C2—H2B	0.9900	C2A—H2A2	0.9900
C3—C4	1.521 (9)	C3A—C4A	1.518 (8)
C3—H3	1.0000	C3A—H3A	1.0000
C4—H4A	0.9900	C4A—H4A1	0.9900
C4—H4B	0.9900	C4A—H4A2	0.9900
C5—C51	1.472 (10)	C5A—C51A	1.465 (10)
C5—H5	0.9500	C5A—H5A	0.9500
C11—C16	1.393 (9)	C11A—C12A	1.394 (9)
C11—C12	1.407 (9)	C11A—C16A	1.397 (9)
C12—C13	1.395 (10)	C12A—C13A	1.371 (9)
C12—H12	0.9500	C12A—H12A	0.9500
C13—C14	1.413 (10)	C13A—C14A	1.400 (9)
C13—H13	0.9500	C13A—H13A	0.9500
C14—C15	1.415 (9)	C14A—C15A	1.400 (9)
C15—C16	1.377 (9)	C15A—C16A	1.386 (9)
C15—H15	0.9500	C15A—H15A	0.9500
C16—H16	0.9500	C16A—H16A	0.9500
C17—H17A	0.9800	C17A—H17D	0.9800
C17—H17B	0.9800	C17A—H17E	0.9800
C17—H17C	0.9800	C17A—H17F	0.9800
C18—H18A	0.9800	C18A—H18D	0.9800
C18—H18B	0.9800	C18A—H18E	0.9800
C18—H18C	0.9800	C18A—H18F	0.9800
C51—C52	1.387 (10)	C51A—C56A	1.377 (10)
C51—C56	1.397 (10)	C51A—C52A	1.384 (9)
C52—C53	1.397 (10)	C52A—C53A	1.373 (10)
C52—H52	0.9500	C52A—H52A	0.9500
C53—C54	1.388 (11)	C53A—C54A	1.399 (12)
C53—H53	0.9500	C53A—H53A	0.9500
C54—C55	1.423 (10)	C54A—C55A	1.401 (10)
C55—C56	1.365 (10)	C55A—C56A	1.416 (10)
C55—H55	0.9500	C55A—H55A	0.9500
C56—H56	0.9500	C56A—H56A	0.9500
C57—H57A	0.9800	C57A—H57D	0.9800
C57—H57B	0.9800	C57A—H57E	0.9800
C57—H57C	0.9800	C57A—H57F	0.9800
C58—H58A	0.9800	C58A—H58D	0.9800
C58—H58B	0.9800	C58A—H58E	0.9800
C58—H58C	0.9800	C58A—H58F	0.9800
			
C3—O1—H1	109 (5)	C3A—O1A—H1A	112 (5)
C1—N1—C2	117.5 (6)	C1A—N1A—C2A	117.7 (6)
C5—N2—C4	117.6 (6)	C5A—N2A—C4A	117.7 (6)
C14—N3—C18	121.0 (6)	C14A—N3A—C17A	121.0 (6)
C14—N3—C17	120.6 (6)	C14A—N3A—C18A	121.1 (6)
C18—N3—C17	118.3 (6)	C17A—N3A—C18A	117.9 (6)
C54—N4—C58	121.4 (7)	C54A—N4A—C57A	120.6 (7)
C54—N4—C57	117.5 (7)	C54A—N4A—C58A	119.6 (7)
C58—N4—C57	120.7 (7)	C57A—N4A—C58A	116.2 (6)
N1—C1—C11	122.4 (6)	N1A—C1A—C11A	123.6 (6)
N1—C1—H1B	118.8	N1A—C1A—H1A1	118.2
C11—C1—H1B	118.8	C11A—C1A—H1A1	118.2
N1—C2—C3	110.8 (5)	N1A—C2A—C3A	110.4 (5)
N1—C2—H2A	109.5	N1A—C2A—H2A1	109.6
C3—C2—H2A	109.5	C3A—C2A—H2A1	109.6
N1—C2—H2B	109.5	N1A—C2A—H2A2	109.6
C3—C2—H2B	109.5	C3A—C2A—H2A2	109.6
H2A—C2—H2B	108.1	H2A1—C2A—H2A2	108.1
O1—C3—C4	111.4 (5)	O1A—C3A—C4A	112.6 (5)
O1—C3—C2	111.3 (5)	O1A—C3A—C2A	110.8 (5)
C4—C3—C2	111.7 (5)	C4A—C3A—C2A	110.6 (5)
O1—C3—H3	107.4	O1A—C3A—H3A	107.5
C4—C3—H3	107.4	C4A—C3A—H3A	107.5
C2—C3—H3	107.4	C2A—C3A—H3A	107.5
N2—C4—C3	112.0 (5)	N2A—C4A—C3A	111.3 (5)
N2—C4—H4A	109.2	N2A—C4A—H4A1	109.4
C3—C4—H4A	109.2	C3A—C4A—H4A1	109.4
N2—C4—H4B	109.2	N2A—C4A—H4A2	109.4
C3—C4—H4B	109.2	C3A—C4A—H4A2	109.4
H4A—C4—H4B	107.9	H4A1—C4A—H4A2	108.0
N2—C5—C51	124.1 (6)	N2A—C5A—C51A	126.0 (7)
N2—C5—H5	117.9	N2A—C5A—H5A	117.0
C51—C5—H5	117.9	C51A—C5A—H5A	117.0
C16—C11—C12	117.5 (6)	C12A—C11A—C16A	117.2 (6)
C16—C11—C1	122.9 (6)	C12A—C11A—C1A	121.5 (6)
C12—C11—C1	119.6 (6)	C16A—C11A—C1A	121.3 (6)
C13—C12—C11	120.9 (6)	C13A—C12A—C11A	121.8 (6)
C13—C12—H12	119.6	C13A—C12A—H12A	119.1
C11—C12—H12	119.6	C11A—C12A—H12A	119.1
C12—C13—C14	121.0 (6)	C12A—C13A—C14A	121.0 (6)
C12—C13—H13	119.5	C12A—C13A—H13A	119.5
C14—C13—H13	119.5	C14A—C13A—H13A	119.5
N3—C14—C13	121.7 (6)	N3A—C14A—C15A	120.5 (6)
N3—C14—C15	120.8 (6)	N3A—C14A—C13A	121.9 (6)
C13—C14—C15	117.4 (6)	C15A—C14A—C13A	117.7 (6)
C16—C15—C14	120.5 (6)	C16A—C15A—C14A	120.6 (6)
C16—C15—H15	119.7	C16A—C15A—H15A	119.7
C14—C15—H15	119.7	C14A—C15A—H15A	119.7
C15—C16—C11	122.5 (6)	C15A—C16A—C11A	121.4 (6)
C15—C16—H16	118.7	C15A—C16A—H16A	119.3
C11—C16—H16	118.7	C11A—C16A—H16A	119.3
N3—C17—H17A	109.5	N3A—C17A—H17D	109.5
N3—C17—H17B	109.5	N3A—C17A—H17E	109.5
H17A—C17—H17B	109.5	H17D—C17A—H17E	109.5
N3—C17—H17C	109.5	N3A—C17A—H17F	109.5
H17A—C17—H17C	109.5	H17D—C17A—H17F	109.5
H17B—C17—H17C	109.5	H17E—C17A—H17F	109.5
N3—C18—H18A	109.5	N3A—C18A—H18D	109.5
N3—C18—H18B	109.5	N3A—C18A—H18E	109.5
H18A—C18—H18B	109.5	H18D—C18A—H18E	109.5
N3—C18—H18C	109.5	N3A—C18A—H18F	109.5
H18A—C18—H18C	109.5	H18D—C18A—H18F	109.5
H18B—C18—H18C	109.5	H18E—C18A—H18F	109.5
C52—C51—C56	116.8 (6)	C56A—C51A—C52A	117.8 (7)
C52—C51—C5	119.1 (7)	C56A—C51A—C5A	122.8 (6)
C56—C51—C5	124.0 (6)	C52A—C51A—C5A	119.3 (7)
C51—C52—C53	122.3 (8)	C53A—C52A—C51A	122.7 (7)
C51—C52—H52	118.8	C53A—C52A—H52A	118.6
C53—C52—H52	118.8	C51A—C52A—H52A	118.7
C54—C53—C52	120.1 (7)	C52A—C53A—C54A	120.4 (7)
C54—C53—H53	119.9	C52A—C53A—H53A	119.8
C52—C53—H53	119.9	C54A—C53A—H53A	119.8
C53—C54—N4	121.4 (7)	N4A—C54A—C53A	120.7 (7)
C53—C54—C55	117.8 (7)	N4A—C54A—C55A	121.3 (8)
N4—C54—C55	120.7 (7)	C53A—C54A—C55A	118.0 (7)
C56—C55—C54	120.5 (7)	C54A—C55A—C56A	120.1 (7)
C56—C55—H55	119.7	C54A—C55A—H55A	120.0
C54—C55—H55	119.7	C56A—C55A—H55A	120.0
C55—C56—C51	122.3 (7)	C51A—C56A—C55A	121.0 (7)
C55—C56—H56	118.8	C51A—C56A—H56A	119.5
C51—C56—H56	118.8	C55A—C56A—H56A	119.5
N4—C57—H57A	109.5	N4A—C57A—H57D	109.5
N4—C57—H57B	109.5	N4A—C57A—H57E	109.5
H57A—C57—H57B	109.5	H57D—C57A—H57E	109.5
N4—C57—H57C	109.5	N4A—C57A—H57F	109.5
H57A—C57—H57C	109.5	H57D—C57A—H57F	109.5
H57B—C57—H57C	109.5	H57E—C57A—H57F	109.5
N4—C58—H58A	109.5	N4A—C58A—H58D	109.5
N4—C58—H58B	109.5	N4A—C58A—H58E	109.5
H58A—C58—H58B	109.5	H58D—C58A—H58E	109.5
N4—C58—H58C	109.5	N4A—C58A—H58F	109.5
H58A—C58—H58C	109.5	H58D—C58A—H58F	109.5
H58B—C58—H58C	109.5	H58E—C58A—H58F	109.5
			
C2—N1—C1—C11	178.6 (6)	C2A—N1A—C1A—C11A	177.8 (6)
C1—N1—C2—C3	116.0 (7)	C1A—N1A—C2A—C3A	117.4 (6)
N1—C2—C3—O1	−175.3 (5)	N1A—C2A—C3A—O1A	−174.1 (5)
N1—C2—C3—C4	59.4 (8)	N1A—C2A—C3A—C4A	60.4 (7)
C5—N2—C4—C3	−128.6 (6)	C5A—N2A—C4A—C3A	−127.7 (6)
O1—C3—C4—N2	71.8 (7)	O1A—C3A—C4A—N2A	71.5 (7)
C2—C3—C4—N2	−163.0 (6)	C2A—C3A—C4A—N2A	−163.9 (5)
C4—N2—C5—C51	177.0 (6)	C4A—N2A—C5A—C51A	179.9 (6)
N1—C1—C11—C16	−2.8 (10)	N1A—C1A—C11A—C12A	179.6 (7)
N1—C1—C11—C12	180.0 (7)	N1A—C1A—C11A—C16A	−0.6 (10)
C16—C11—C12—C13	−1.7 (10)	C16A—C11A—C12A—C13A	−4.5 (10)
C1—C11—C12—C13	175.7 (6)	C1A—C11A—C12A—C13A	175.3 (6)
C11—C12—C13—C14	−1.2 (11)	C11A—C12A—C13A—C14A	1.0 (11)
C18—N3—C14—C13	−179.0 (7)	C17A—N3A—C14A—C15A	−178.2 (8)
C17—N3—C14—C13	−3.1 (11)	C18A—N3A—C14A—C15A	−1.3 (11)
C18—N3—C14—C15	3.1 (11)	C17A—N3A—C14A—C13A	2.2 (11)
C17—N3—C14—C15	178.9 (7)	C18A—N3A—C14A—C13A	179.1 (7)
C12—C13—C14—N3	−174.8 (7)	C12A—C13A—C14A—N3A	−177.5 (7)
C12—C13—C14—C15	3.2 (10)	C12A—C13A—C14A—C15A	2.9 (10)
N3—C14—C15—C16	175.8 (7)	N3A—C14A—C15A—C16A	177.2 (7)
C13—C14—C15—C16	−2.2 (10)	C13A—C14A—C15A—C16A	−3.1 (10)
C14—C15—C16—C11	−0.7 (10)	C14A—C15A—C16A—C11A	−0.4 (10)
C12—C11—C16—C15	2.7 (10)	C12A—C11A—C16A—C15A	4.2 (10)
C1—C11—C16—C15	−174.6 (6)	C1A—C11A—C16A—C15A	−175.6 (6)
N2—C5—C51—C52	169.0 (7)	N2A—C5A—C51A—C56A	−9.9 (11)
N2—C5—C51—C56	−14.9 (11)	N2A—C5A—C51A—C52A	173.4 (7)
C56—C51—C52—C53	−0.3 (11)	C56A—C51A—C52A—C53A	0.3 (10)
C5—C51—C52—C53	176.0 (7)	C5A—C51A—C52A—C53A	177.1 (7)
C51—C52—C53—C54	−1.0 (12)	C51A—C52A—C53A—C54A	−0.3 (11)
C52—C53—C54—N4	−176.9 (7)	C57A—N4A—C54A—C53A	8.3 (10)
C52—C53—C54—C55	0.7 (11)	C58A—N4A—C54A—C53A	166.3 (7)
C58—N4—C54—C53	−175.5 (7)	C57A—N4A—C54A—C55A	−171.8 (7)
C57—N4—C54—C53	−2.9 (11)	C58A—N4A—C54A—C55A	−13.9 (11)
C58—N4—C54—C55	7.0 (12)	C52A—C53A—C54A—N4A	−179.6 (7)
C57—N4—C54—C55	179.6 (7)	C52A—C53A—C54A—C55A	0.6 (11)
C53—C54—C55—C56	1.1 (11)	N4A—C54A—C55A—C56A	179.4 (6)
N4—C54—C55—C56	178.6 (7)	C53A—C54A—C55A—C56A	−0.8 (11)
C54—C55—C56—C51	−2.5 (12)	C52A—C51A—C56A—C55A	−0.5 (10)
C52—C51—C56—C55	2.1 (11)	C5A—C51A—C56A—C55A	−177.2 (6)
C5—C51—C56—C55	−174.0 (7)	C54A—C55A—C56A—C51A	0.8 (11)

### Hydrogen-bond geometry (Å, º)

**Table:**

*D*—H···*A*	*D*—H	H···*A*	*D*···*A*	*D*—H···*A*
O1—H1···N2*A*^i^	0.84 (8)	2.06 (8)	2.889 (7)	170 (7)
O1*A*—H1*A*···N2	0.84 (8)	2.04 (8)	2.863 (7)	166 (7)
C57—H57*B*···O1^ii^	0.98	2.53	3.171 (9)	123
C57*A*—H57*E*···O1*A*^iii^	0.98	2.36	3.211 (8)	145

Symmetry codes: (i) *x*−1, *y*, *z*; (ii) −*x*, *y*−1/2, −*z*; (iii) −*x*+1, *y*−1/2, −*z*.
